# Ownership and utilization of bed nets and reasons for use or non-use of bed nets among community members at risk of malaria along the Thai-Myanmar border

**DOI:** 10.1186/s12936-021-03837-5

**Published:** 2021-07-06

**Authors:** Kasama Pooseesod, Daniel M. Parker, Natthani Meemon, Saranath Lawpoolsri, Pratap Singhasivanon, Jetsumon Sattabongkot, Liwang Cui, Suparat Phuanukoonnon

**Affiliations:** 1 Department of Tropical Hygiene, Faculty of Tropical Medicine, Mahidol University, Nakhon Pathom, Thailand; 2 Faculty of Public Health, Thammasat University, Bangkok, Thailand; 3 Department of Population Health & Disease Prevention, Program in Public Health Susan and Henry Samueli College of Health Sciences, University of California, Irvine, USA; 4 Department of Society and Health, Faculty of Social Sciences and Humanities, Mahidol University, Nakhon Pathom, Thailand; 5 Mahidol Vivax Research Unit, Faculty of Tropical Medicine, Mahidol University, Nakhon Pathom, Thailand; 6 Department of Internal Medicine, Morsani College of Medicine, University of South Florida, Tampa, FL USA; 7 Faculty of Tropical Medicine, SEAMEO TROPMED Regional Centre for Tropical Medicine, Mahidol University, Nakhon Pathom, Thailand; 8 Department of Social and Environmental Medicine, Faculty of Tropical Medicine, Mahidol University, Nakhon Pathom, Thailand

**Keywords:** Malaria, Bed net, Long-lasting insecticidal net, Karen ethnic, Forest goers, Thailand

## Abstract

**Background:**

With the goal for malaria elimination in Thailand set for 2024, increased coverage and utilization of bed net, especially insecticide-treated net (ITN) or long-lasting insecticidal net (LLIN) is a key strategy. This study aims to provide the necessary information about bed net ownership and utilization among the population at risk of malaria living along the Thai-Myanmar border in Tak province.

**Methods:**

A cross-sectional study was conducted using a mixed-method approach in 331 households from 5 hamlets in the villages of the Thai-Myanmar border. The research tools included a questionnaire, bed net inspection, and semi-structured interviews. Logistic regression was used to explore the sociodemographic factors associated with bed net utilization. The qualitative analysis employed a thematic analysis approach.

**Results:**

This survey found that 98.5% of households had at least one bed net per household, and 74.3% had at least one ITN/LLIN. However, only 30.8% of households reached the standard policy set by the Minister of Public Health of one ITN/LLINs per two persons. Most residents used bed net (92.1% used in the previous night and 80.9% used every day). For those using bed nets, however, 61.9% used ITNs or LLINs the night before and 53.1% used them every day. Nonetheless, the usage rates of bed nets (any type) in the previous night among children and pregnant women were high, reaching 95.3% and 90.0%, respectively. Seven explanatory variables showed statistically significant associations with bed net use every day, including: “not staying overnight in the forest or the field”, “sleeping pattern based on gender”, “sufficient numbers of bed nets to cover all sleeping spaces”, “preference for free bed nets”, “age”, “gender”, and “SES score” showed statistically significant association with bed net use every day. The major reasons for the regular use of bed nets in both household and the forest were to prevent mosquito biting. The reasons for not using bednets in the household were discomfort feelings from heat, perception of unnecessity due to low mosquito density, whereas the reason for not using bed nets in the forest was inconvenience.

**Conclusion:**

Despite that overall coverage and usage of bed nets was high, only one third reached the standard level specified by the policy. Overnight in the forest, the dissatisfaction with the quality of free bed nets, insufficient number of bed nets, sleeping alone, male gender, age more than 10 years, low socioeconomic status, discomfort from heat, perception of no benefits of bed nets due to low mosquito density, and inconvenience were factors influencing bed net use. Maintaining high coverage and utility rate of bed nets should be a priority for the malaria high-risk population.

**Supplementary Information:**

The online version contains supplementary material available at 10.1186/s12936-021-03837-5.

## Background

Based on the 2020 world malaria report, there were 87 countries and areas with ongoing malaria transmission and approximately 229 million malaria cases in 2019 [1].Thailand reported a substantial decline (19%) in total reported cases between 2018 and 2019 [2]. Malaria in Thailand is patchy in its distribution and can be typified as ‘border malaria’ and ‘forest malaria’, with the highest transmission along international borders and in rural forested areas [3]. The western border with Myanmar has had the highest burden of malaria and has been the focus of malaria control programmes for decades [3]. Motivated by the continuous decrease in malaria burden, the Thai government has declared a national malaria elimination plan intending to achieve this by the year 2024 [4].

The major malaria vectors in Thai-Myanmar border include *Anopheles minimus *sensu lato (*s.l*.) (40.32%) and *Anopheles maculatus s.l.* (21.43%) [5]. *Anopheles minimus* was most abundant during the transition from wet to dry season and found more indoor than outdoor. *Anopheles maculatus* was the most abundant during the wet season in both indoor and outdoor. Both species are typically found in or near hilly, forested regions [6, 7]. Moreover, *Anopheles annularis s.l.* and *Anopheles barbirostris s.l.* were also identified as additional vectors with potential outdoor malaria transmission after the wet season [5]. The potential roles of different anopheline species in human malaria transmission in this region have been the challenge for effective vector control.

To accomplish this elimination goal within the time frame, a key strategy is to increase the bed net coverage and utilization, especially insecticide-treated nets (ITN) or long-lasting insecticidal nets (LLIN). The goal is to achieve 90% LLIN coverage among populations in high transmission areas (designated as A1 or A2 areas). A1 is village with reported indigenous malaria cases in current financial year and A2 is village without indigenous malaria cases for past 1–3 years [8]. Previous studies among the general population in Thailand and among the populations on the Thai-Myanmar border (Prachuap Khiri Khan Province) identified poor coverage and poor utilization of ITN/LLINs [9, 10]. However, actual coverage and utilization of bed nets as well as factors attributing to bed net use have not been investigated in this study area.

The objective of this study was to assess the ownership, accessibility, and utilization of both treated (ITNs and LLINs) and untreated bed nets in a remaining malaria transmission focus in western Thailand. A cross-sectional study was conducted and questionnaires were used to determine predictors of bed net use. The results are useful for formulating appropriate policies for the control programmes and for the promotion of LLINs and long-lasting insecticide-treated hammock net (LLIHN) within the context of populations living along the malarious borders.

## Methods

### Study design

This is a mixed-methods cross-sectional study that included a questionnaire, an inspection form, and semi-structured interviews among selected participants. The household survey was conducted from August to October 2019.

### Study site

**Fig. 1:**
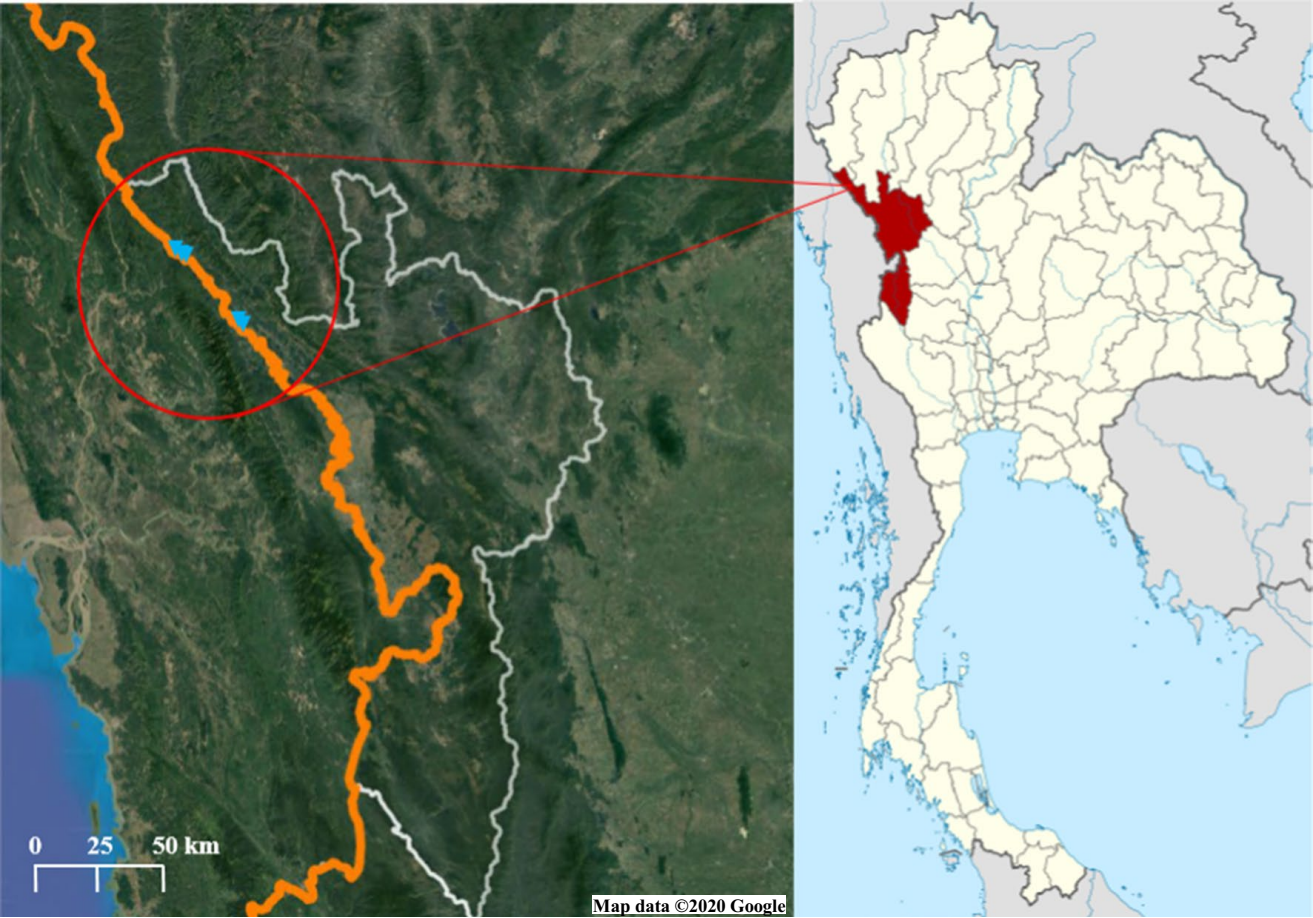
The study site: Tha Song Yang district, Tak province, Thailand [72, 73]

A community-based cross-sectional survey was conducted in the areas under the International Center of Excellence for Malaria Research (ICEMR) project in Tha Song Yang District, Tak Province, northwestern Thailand (Fig. 1). Tha Song Yang is situated in the northwestern region of Tak, on the Moei River bank near the Myanmar border. The climate is tropical with an annual average temperature of 26.4 °C. The rainy season is between May and October, with an average yearly rainfall of 1540 mm. The inhabitants of this area are approximately 30% Thai and 70% ethnic minorities. Normally, there are two peaks of malaria transmission, one at the beginning of the rainy season (May–August) and the other at the end of the rainy season (October) [11]. *Plasmodium vivax* and *Plasmodium falciparum* are the predominant species in this region, although all human malaria parasites, as well as the simian malaria species *Plasmodium knowlesi*, have been identified in this area [12]. This study was conducted in 5 hamlets including Nong Bua, and Tala Oka (Mae Usu sub-district), Suan Oi, Pha Man, and Ko Ma Nae (Tha Song Yang sub-district).

### Study population, sampling and sample size

**Fig. 2:**
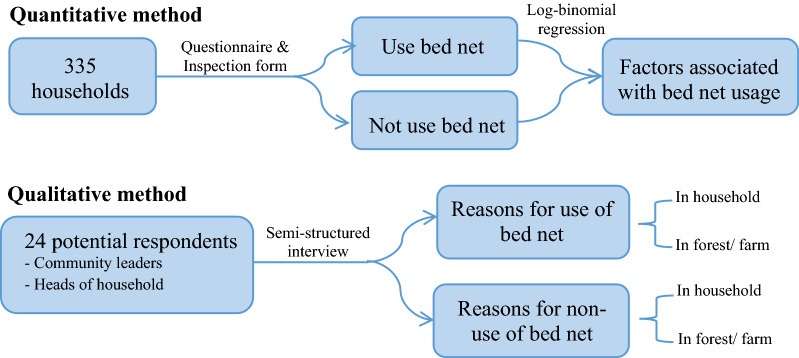
Schematic diagram of the study

For the quantitative component of the study, households residing in the study site were randomly selected from the study villages. Out of a total of 918 households in the 5 hamlets, 335 were selected by probability proportional to hamlet size, using an online statistical calculator [13]. Most of the household members are the Karen ethnic group [14]. From each household, the questionnaire and inspection form were administered to heads of household or representatives who look after the household. For the qualitative component, 24 potential respondents including 2 community leaders from each sub-district, and 4 heads of household from each hamlet were selected to participate in the semi-structured interview. Purposive sampling was used to recruit participants for this part of the research. The target participation and the potential participants were selected based on geography, age, gender, and reported bed net use (including both those who do and do not report using bed nets). Important community leaders as “gatekeepers” were consulted and engaged in order to identify and invite the participants to join this study. Schematic diagram of the study was illustrated in Fig. 2.

### Household surveys

A community-based survey was conducted to assess the ownership, accessibility, and utilization of both treated (ITNs and LLINs) and untreated bed nets using a questionnaire, inspection form, and semi-structured interviews. Respondents from 331 households in completed the interviews. In the selected households, face-to-face interviews were conducted with heads of household by a trained study team who speak and understand both Thai and Karen languages.

### Data analysis and statistics

Data were extracted from the survey database and imported into the SPSS program version 22.0 [15] for analysis. Proportions (with 95% confidence intervals) were used to summarize categorical variables related to ownership, accessibility, and utilization of bed nets. A single variable for socioeconomic status (SES) was generated using factor analysis of mixed data (FAMD), using a combinatoin of continuous and categorical variables (full list in Additional file 1). Logistic regression was then used to explore the sociodemographic factors associated with bed net utilization (1 = yes, used bed net; 0 = no, did not use). The logistic regression model included a random intercept for household to account for confounding and difference in response variation within and between households. Adjusted odds ratios (AOR) (with 95% confidence intervals [CIs]) were used to measure the strength of this association.

The qualitative analysis employed a thematic analysis approach [16]. The tentative code categories were given in the conceptual framework. Data were analysed using thematic analysis of content to allow bringing together of similar views from different respondents together.

## Results

### Household-level characteristics

**Fig. 3:**
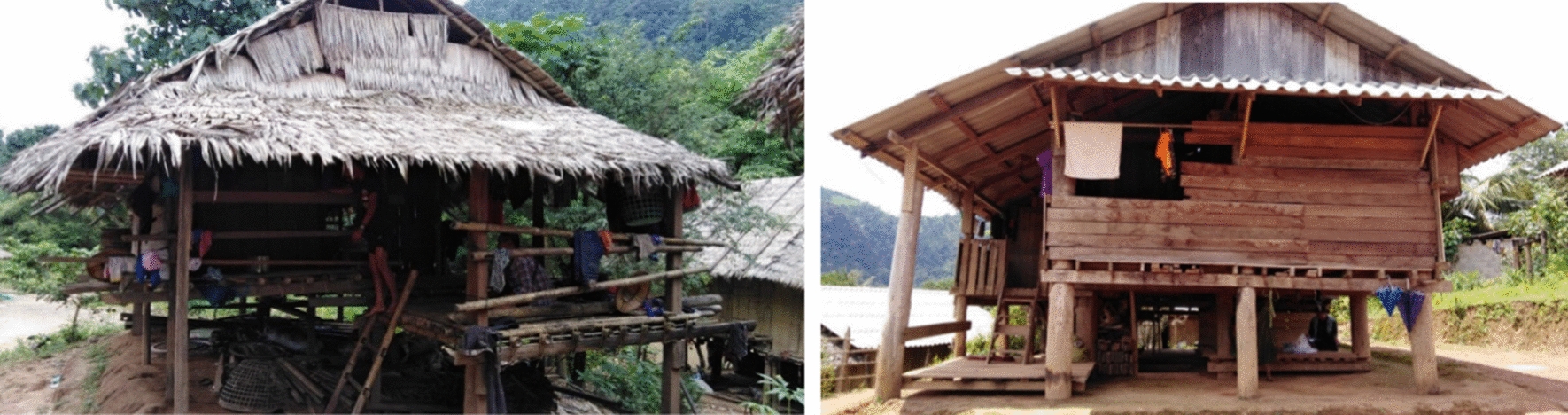
Housing characteristics of the border population in Tha Song Yang district, Tak province, Thailand

For the 331 households participating in the study, the majority had bamboo/wood walls (94.56%), bamboo/wood floors (82.18%), and terracotta/galvanized iron roofs (71.60%) (Fig. 3). Among them, 315 (95.17%) households were from the Karen ethnic minority, and 4.83% were Thai. Questionnaires were administered to 122 (36.86%) male and 209 (63.14%) female household heads. The mean age of the 331 respondents was 43.42 (± 14.03) years; 229 (69.18%) heads of households were 35 years or older. A large proportion (77.64%) of the respondents were illiterate. Over half (54.98%) of the households had a family income of ≤ 63.80 USD/month (Table 1). For the 1,423 household members who reported bed net use, 787 (55.31%) were female (Table 2). The mean age of the household members was 25.68 (± 20.05) years; 620 (43.57%) were 18–59 years old. For the participating household members, 186 (13.07%) of whom stayed in the forests or the field.

**Table 1:** Household and heads of household characteristics of border population in Tha Song Yang district, Tak province, Thailand

Characteristics	Total n = 331, n (%)
Wall	
Bamboo/wood	313 (94.56)
Concrete	18 (5.44)
Floor	
Bamboo/wood	272 (82.18)
Concrete	59 (17.82)
Roof	
Thatch	94 (28.40)
Terracotta/galvanized iron	237 (71.60)
Sex	
Male	122 (36.86)
Female	209 (63.14)
Age (years)	
< 35	102 (30.82)
≥ 35	229 (69.18)
Mean (± SD)	43.42 (± 14.03)
Ethnics	
Thai	16 (4.83)
Karen	315 (95.17)
Education level	
Illiterate	257 (77.64)
Literate	74 (22.36)
Family income/month (Bath)	
≤ 2000	182 (54.98)
> 2000	149 (45.02)
Mean (± SD)	2630.51 (± 1321.23)

**Table 2:** Household member characteristics of border population in Tha Song Yang district, Tak province, Thailand

Characteristics	Total n = 1423, n (%)
Sex	
Male	636 (44.69)
Female	787 (55.31)
Age (years)	
≤ 10	428 (30.08)
11–17	263 (18.48)
18–59	620 (43.57)
≥ 60	112 (7.87)
Mean (± SD)	25.68 (± 20.05)
Household member stay overnight outside in the forest or the field	
Yes	186 (13.07)
No	1237 (86.93)

### Bed net ownership, access, and utilization

Table 3 shows the household-level bed net ownership, access, and utilization. Almost all (98.49%) households had at least one bed net per household, and 74.32% had at least one ITNs/LLINs in the household. However, only 30.82% of all households had sufficient numbers of ITN/LLINs, as per standard policy i.e., one ITN/LLIN per two persons. Moreover, only 4.30% of forest goers had owned LLIHNs. Besides, 86.10% of the households had sufficient numbers of bed net to cover all sleeping spaces. Although 94.17% of the household members slept in the sleeping spaces with any type of bed nets, 63.46% slept in sleeping spaces with ITNs or LLINs.

**Table 3:** Household ownership access and utilization of bed nets among border population in Tha Song Yang district, Tak province, Thailand

Characteristics	n (%)
Household ownership of bed nets	
At least one net per household (any type)	326 (98.49)
At least one ITN/LLIN per household	246 (74.32)
Access of bed nets	
One net per two people (any type)	168 (50.76)
One ITN/LLIN per two people	102 (30.82)
One LLIHN per one people	8 (4.30)
% of population with access to any bed net	1340 (94.17)
% of population with access to ITN/LLIN	903 (63.46)
% of household have sufficient numbers of bed net to cover all sleeping spaces	285 (86.10)
Utilization of bed nets	
% of population slept under any bed net last night	1311 (92.13)
% of population slept under an ITN/LLIN last night	881 (61.91)
% of forest goers slept under an ITN/LLIN last night	122 (65.59)
% of population slept under any bed net every day	1151 (80.89)
% of population slept under an ITN/LLIN every day	756 (53.13)
% of forest goers slept under an ITN/LLIN every day	16 (8.60)
% of children under 10 years slept under any bed net last night	408 (95.33)
% of children under 10 years slept under an ITN/LLIN last night	255 (59.58)
% of pregnant women slept under any bed net last night	10 (100.00)
% of pregnant women slept under an ITN/LLIN last night	9 (90.00)

Most of the household members (92.13%) reported using bed nets in the previous night, and 881 (61.91%) used ITNs or LLINs (Table 3). Since some respondents did not use bed net every day, especially in the summer season or when staying overnight in the forests or agricultural fields, daily use of bed net was also recorded. While 1,151 (80.89%) household members reported bed net usage every day, 756 (53.13%) used ITNs or LLINs. Only 8.60% of forest goers use ITN/LLIN every day. For children under 10 years (n = 428), 95.33% slept under a bed net in the previous night, and 59.58% slept under an ITN/LLIN. Among the pregnant women (n = 10), this was 100% and 90%, respectively. Out of all 601 inspected bet nets, 109 (18.14%) had holes (defined as a tear or opening that a finger could fit through). For the age of bed net, 52.08% of the bed nets were up to 1 year old**,** 44.59% were 2–3 years old**,** and 3.33% were more than 3 years old.

The most common pattern of bed net deployment by the household heads included inspecting for holes (86.32%) and checking for mosquitoes trapped inside (83.06%). However, only 36.81% tucked in the nets fully before sleeping, and 28.99% slept away from the edges of the bed. Only 103 (33.55%) of the household heads deployed the bed nets properly (Table 4).

**Table 4:** Pattern of deployment of bed net by head of household owning and using bed net (n = 307)

	n	%
Pattern of deployment of bed nets^a^		
Tucking in fully during sleeping	113	36.81
Inspecting for holes	265	86.32
Checking for mosquitos trapped inside	255	83.06
Sleeping away from the edge of the bed	89	28.99
Head of household deployed bed net properly		
Yes	103	33.55
No	204	66.45
Total	307	100.00

^a^Multiple responds allowed

### Sociodemographic factors associated with bed nets usage

Univariate logistic regressions were to compare the daily bed nets use across the different variables. Based on the univariate models, strong associations were found between bed net use and stay overnight in the forest or the field [no *versus* yes: OR = 67.80, 95% CI = 42.77–107.49; p < 0.001], sleeping pattern based on same gender [female sleep with female vs male sleep alone: OR = 15.94, 95% CI = 7.00–36.27; p < 0.001], sufficient numbers of bed net to cover all sleeping spaces [yes *versus* no: OR = 6.03, 95% CI = 4.28–8.48; p < 0.001], preference for free bed net [yes or indifferent *vs* not like: OR = 5.50, 95% CI = 3.36–9.02; p < 0.001], and age [18–59 *vs* ≤ 10: OR = 0.08, 95% CI = 0.04–0.14; p < 0.001]. Additionally, a weak association was found between daily bed net use and marital status [others *vs* married: OR = 3.62, 95% CI = 2.70–4.87; p < 0.001], gender [female *vs* male: OR = 3.20, 95% CI = 2.39–4.28; p =  < 0.001], sleeping pattern based on younger age (≤ 10 year) [sleep with child *vs* not sleep with child: OR = 2.65, 95% CI = 2.00–3.52; p < 0.001]. However, SES score was not significantly associated with bed net use.

In the final multivariable logistic regression, seven explanatory variables including: “not staying overnight in the forest or the field”, “sleeping pattern based on gender”, “sufficient numbers of bed net to cover all sleeping spaces”, “preference for free bed net”, “age”, “gender”, and “SES score” showed a significant association with daily bed net use (Table 5).

**Table 5:** Variables related to use of bed nets every day among respondents living in households owning at least one net (n = 1401)

	n (%)	Univariate analysis		Multivariable analysis	
		OR (95% CI)	p-value	OR (95% CI)	p-value
Age of household member (years)					
≤ 10 (n = 418)	405 (96.89)	1	–	–	–
11–17 (n = 259)	220 (84.94)	0.17 (0.09–0.33)	< 0.001**	0.17 (0.07–0.43)	< 0.001**
18–59 (n = 613)	442 (72.10)	0.08 (0.04–0.14)	< 0.001**	0.13 (0.04-.36)	< 0.001**
≥ 60 (n = 111)	84 (75.68)	0.09 (0.04–0.18)	< 0.001**	0.24 (0.07–0.85)	0.027*
Gender of household member					
Male (n = 625)	456 (72.96)	1	–	–	–
Female (n = 776)	695 (89.56)	3.20 (2.39–4.28)	< 0.001**	2.44 (1.41–4.22)	0.001*
Marital status of household member					
Married (n = 610)	439 (71.97)	1	–	–	–
Others (n = 791)	712 (90.01)	3.62 (2.70–4.87)	< 0.001**	0.66 (0.29–1.47)	0.304
Household member stay overnight outside in the forest or the field					
Yes (n = 184)	27 (14.67)	1	–	–	–
No (n = 1,217)	1124 (92.36)	67.80 (42.77–107.49)	< 0.001**	96.25 (52.17–177.58)	< 0.001**
Head of household prefer to use free bed net					
Not prefer (n = 75)	34 (45.33)	1	–	–	–
Yes or indifferent (n = 1,326)	1117 (84.24)	5.50 (3.36–9.02)	< 0.001**	13.42 (6.47–27.85)	< 0.001**
Sufficient numbers of bed net to cover all sleeping spaces in household					
No (n = 190)	104 (54.74)	1	–	–	–
Yes (n = 1,211)	1047 (86.46)	6.03 (4.28–8.48)	< 0.001**	7.22 (4.29–12.14)	< 0.001**
Sleeping pattern based on gender					
Male sleep alone (n = 98)	61 (62.24)	1	–	–	–
Female sleep alone (n = 75)	63 (84.00)	3.34 (1.58–7.06)	0.002*	1.00 (0.33–3.06)	0.998
Male sleep with female (spouse) (n = 162)	104 (64.20)	1.18 (0.70–2.00)	0.536	1.53 (0.57–4.10)	0.399
Male sleep with male (≥ 2 person/sleeping space) (n = 57)	43 (75.44)	2.17 (1.04–4.55)	0.040*	1.41 (0.43–4.59)	0.568
Male sleep with female (not spouse) (≥ 2 person/sleeping space) (n = 808)	687 (85.02)	4.00 (2.51–6.35)	< 0.001**	2.88 (1.13–7.37)	0.027*
Female sleep with female (≥ 2 person/sleeping space) (n = 201)	193 (96.02)	15.94 (7.00–36.27)	< 0.001**	4.42 (1.27–15.47)	0.020*
Sleeping pattern based on age (≤ 10 year)					
Not sleep with child ≤ 10 year (n = 533)	394 (73.92)	1	–	–	–
Sleep with child ≤ 10 year (n = 868)	757 (87.21)	2.65 (2.00–3.52)	< 0.001**	0.72 (0.35–1.47)	0.370
SES score					
x̄ ± SD	0.07 ± 0.11	2.16 (0.53–8.76)	0.282	13.93 (2.07–93.88)	0.007*

^*^p-value < 0.05, **p-value < 0.001

### Reasons for use or non-use of bed nets in the household and the forest

A total of 22 respondents were interviewed by semi-structured interview. The results organized into key themes that emerged from the discussions.

#### Reasons for use of bed nets in the household

Most participants reported using bed nets to prevent mosquito bites and malaria infection. Most of them remembered using bed nets from a very young age, whereas some of them started to use bed nets when they had children or grandchildren. Bed net use had become a habit for them and they could not sleep without it.

“I use bed nets to protect myself against mosquito bites. I have used bed nets since I was born and becoming my habit I cannot sleep without the net” (Female villager, Mae Usu subdistrict).

Additionally, free bed net distribution campaigns were mentioned as a factor that supported them to start and continue using bed nets in the last 20–30 years.

“After I immigrated to Thailand 20 years ago, I started to use bed nets and they were free bed nets distributed from health providers. The net is good and prevents mosquitoes from biting and not getting sick with malaria.” (Female villager, Tha Song Yang subdistrict).

#### Bed net use in farms and forests

Forest goers in this area had not used any kinds of bed net while they were in the forest. Most forest goers used bed net only in the subsistence farm huts, their temporary residence nearby the forest. Both forest goers and farmers, if they had sufficient bed nets for use in their households, they would take the old bed nets for use in the subsistence farm huts. If they had limited bed nets, they would carry bed net to the subsistence farm hut and carry it back home, especially when children accompanied them to the farms. However, many of them used bed nets in a subsistence farm hut only in the rainy season when mosquitoes are abundant.

“When children accompanied us to the farm plots or forest, we always carried bed net to use in the farm hut. Our children need to get protection from mosquito bites.” (Female villager, Mae Usu subdistrict).

“I sleep under bed net every day. And even when I was going to overnight in the farm, I took an old bed net to use in the farm hut.” (Male villager, Tha Song Yang subdistrict).

#### Reasons for non-use of bed nets in the household

The major reason that participants reported for not using bed nets every day at home was discomfort from the heat and perception of unnecessity due to low mosquito density, especially in the hot season.

“I am still afraid of malaria, but I could not sleep it was too hot to sleep under the nets in the summer” (Female villager, Tha Song Yang subdistrict).

The second most commonly reported reason was feeling complacent and not wanting to expend the effort needed to set up and use nets in the evenings, especially for adolescents who did not share sleeping space with their parents.

“My son and daughter rarely use bed net because they are so lazy to hang the bed net up. I sometimes have to hang it for them and tell them to sleep under bed net.” (Female villager, Mae Usu subdistrict).

Other reasons mentioned for not using a net at home included inadequate number of bed nets, being not habitual of sleeping under the bed nets, use of alternative mosquito control methods, too small size of the free bed net, rough texture of material of free bet nets, strong smell of insecticide, and inadequate space to hang a bed net.

#### Reasons for non-use of bed nets in the forest or farms

The major reason for the non-use of bed nets in the forest was inconvenient to carry the bed nets and prioritized other essential items. Even in the subsistence farm hut, some felt lazy to unpack and hang the bed nets up before sleeping in. Although someone received an LLIHN to use in the forest, they tried to use the hammocks in the forest, they found problems from using the net.

“Going to forest, we travel light with a few necessary items so we can carry lots forest products when we return. It is inconvenient to bring the net to the forest, it takes space to carry. It also can get entangled with other items, obstructed hunting and gatering process, in particular to hunting we need to be quick to shoot the targets. Sometime at night, animals such as elephants approach our sleeping area, we have to run away, we have no time to pack the net.” (Male villager, Tha Song Yang subdistrict).

The second reason was the inadequate number of the nets for use when visiting the forest. Other reasons for not using a net in the forest included being not habitual of sleeping under the bed nets, discomfort from heat, and use of alternative mosquito control methods.

## Discussion

This is the first cross-sectional study assessing the ownership and utilization of bed nets among the border population in Tha Song Yang District of western Thailand. From the result, bed nets are widely available in these localities, a result of efforts by the Department of Disease Control and other partners (such as non-governmental organizations) that have distributed free LLINs from the Global Fund [9]. However, some households did not have ITNs/LLINs. These results are consistent with the finding among the general population in Thailand and among the populations on the Thai-Myanmar border (Prachuap Khiri Khan Province) showed high ownership of bed nets, but poor coverage of ITNs [9, 10]. Similar studies in Myanmar showed high coverage of bed nets with coverage of ITN/LLIN among households of migrant population [17, 18].

This study showed only one third households had sufficient numbers of ITN/LLIN for one ITN/ LLINs per two persons. This was below the standard level of WHO recomendations for universal coverage of ITN/LLINs [19]. This insufficiency might be due to the insufficient number of LLINs obtained from the MOPH and operational challenges of the LLIN distribution system, as some studies identified operational barriers to LLIN distribution [20, 21]. This study suggests that the performance of LLIN distribution system and the operational challenges of LLIN distribution in Thailand should be evaluated, and the insufficiency of ITN/LLIN is also a concern and should be used as an indicator for the LLIN distribution programme. A study in Mozambique reported that the access indicators of LLINs were high when the bed net distribution campaigns used a novel distribution model and used LLINs designated for each sleeping space [22].

Globally, malaria-related morbidity and mortality are highest in children and pregnant women [23]. Overall bed net usage among adults in this study was lower than those among children and pregnant women. This was mainly due to the high focus on vulnerable groups. Sleeping with a female household member (i.e. father with daughter, mother with son) was also associated with higher bed net usage when compared with males sleeping alone. However, approximately a half of the children in this study used ITNs/LLINs, consistent with the previous findings in Myanmar and some countries in Africa which showed that the utilization of ITN remained moderate or low among children. [17, 18, 24, 25], One of the reasons for low untilization were insufficiency of ITN/LLIN among migrant of Myanmar [17, 18]. Conversely, the factors significantly associated with children ITNs utilization in Ethiopia and Ghana were caretaker’s age being < 30 years, small family size (≤ 5 members), and more of sleeping spaces (≥ 2) [24, 25]. Pregnant women, particularly primigravidas with malaria, have a high risk of severe malaria and low birth weights [26, 27]. This study showed a high rate of ITN/LLIN use among pregnant women. However, in many parts of the world, ITN usage remained moderate or low among pregnant women [18, 28–31].

Most bed nets effectively protect against mosquitoes within three years of WHO’s recommended use [32]. Nonetheless, to increase the effective protection, bed nets need regular re-impregnation with insecticide and get holes repaired. Nets may be less effective if not deployed properly such as tucking in properly or being lifted multiple times over the course the night [33]. Despite providing health education during LLIN distribution (head of the Vector-Borne Disease Unit 2.3.6. personal communication), most people still did not deploy bed nets properly and some did not use the nets every day. This finding suggests the need for new motivational approaches or tools to encourage proper use of nets and using bed nets every day.

One of major factors in bed net use was net manufacturing materials and size affecting net utilization among households with more family members [10]. Many other studies also reported under usage of nets due to the insufficient nets for all household members [10, 17, 34, 35]. Household socio-economic status has likewise been shown to influence bed net access and is the strongest determinant of net use [30, 36–43]. This study showed that wealthier families were significantly more likely to use bed nets every day than the poorer families, likely because of the ability to purchase bed nets.

Consistent with previous studies [33, 44–46], the perceived role of bed nets as a means to protect against mosquitoes and malaria was a significant predictor of seasonal bed net use. In this study, participants reported higher bed net usage during the rainy season. Aside from malaria prevention, the main reason for sleeping under nets was because they provided comfortable sleep and protected against biting insects (including mosquitoes).

Other reasons for the not using bed nets were in line with other research [10, 18, 47–52] suggesting that discomfort from heat and low mosquito density are common reasons for non-adherence. Complacence, the inadequate number of of bed net, rough materials of bed net, not having a routine of bed net use, use of alternative mosquito control methods, and too small size of free bed nets were the other most commonly cited reasons for not using a net at home. Some studies have shown that bed nets decrease airflow, making it feel hot and stifling under a net [53, 54]. In seasons of low and/or variable mosquito nuisance, education will need to emphasize that the risk of malaria is not necessarily diminished when mosquito numbers are perceived to be low [55].

Work and evening activities in the forest affected where community members choose to sleep on any particular evening. Together with the previous findings in Prachuab Khiri Khan province [10] and Southern Thailand [56], sleeping elsewhere especially at temporary shelters in the plantation was main non-use reason. The reasons for not using bed net in the forest were also consistent with studies in the Greater Mekong Subregion [57–59]. Some forest-goers described the inconvenience of carrying bed nets, while others found inadequacy of nets for use when visiting the forest. This study suggests that the campaign of LLIHN distribution appeared not effective for malaria prevention among forest goers. The advantages of these nets might be not adequately promoted as well as other strategies to address outdoor transmission may need to be considered among this population [60–62].

Previously reported limited effectiveness of bed nets in Southeast Asia may have resulted from the vector feeding behaviour and human activities that in some circumstances increase human-vector contacts [63, 64]. Some main malaria vectors, such as *An. minimus*, *An. maculatus* and *Anopheles dirus* feed outdoors at dusk between 6 and 7 p.m. when people are not in bed [63, 65]. Also, forest goers exhibit behaviors (hunting, gathering or fishing) that reduce the protection of bed nets (ITNs) at peak biting times [66, 67]. Nevertheless, given the broader impacts of ITNs for preventing all vector-borne diseases, such as malaria [68, 69], Japanese encephalitis [70], and leishmaniasis [71], proper net distribution and encouraged use still need to be strengthened, especially for the high-risk populations.

For the study limitations, this study was conducted in Thasongyang district; therefore, the results could represent population living along Thai-Myanmar border and Karen ethnic groups, but not represent Karen groups from other areas in which lifestyle, culture, or health operation were different. Due to the limitation of budget and time, this study was conducted in one season (rainy season with more abundant of mosquitoes) which may result in high rate of bed net use in the previous night. Nonetheless, adding question of everyday use of bed net, should reduce the bias from season differences. Recall bias may exist, especially with the questions regarding actual age of bed nets; some respondents could not recall the distribution date or purchase date. This study did not assess the impact of bed nets on malarial infections, therefore; furture research needs to address this aspect.

## Conclusions

This study showed that high overall coverage and usage of bed nets in the study area; however, only one third reached the standard level specified by the policy. Overnighting in the forest or the farm plots, the dissatisfaction with the quality of free bed nets, insufficient number of bed nets, sleeping alone, male gender, age more than 10 years, low socioeconomic status, discomfort from heat, perception of no benefits of bed nets due to low mosquito density, and inconvenience were factors influencing bed net use. Moreover, only one third of heads of households deployed bed net properly. Further health promotion programmes should ensure to maintain high coverage and utility rate of bed nets and further studies should prioritize the investigation of new preventive tools for effective outdoor protection in particualt ot the forest goers.

## Supplementary Information

**Additional file 1:** The list of socioeconomic status (SES) variables.

## Data Availability

The datasets used and/or analysed during the current study are not provided.

## Abbreviations

ITN: Insecticide-treated net
LLIN: Long-lasting insecticidal net
LLIHN: Long-lasting insecticide-treated hammock net
ICEMR: International Center of Excellence for Malaria Research
PR: Prevalence ratio
CI: Confidence interval
