# Supplementary material for: Ownership and utilization of bed nets and reasons for use or non-use of bed nets among community members at risk of malaria along the Thai-Myanmar border
Source: Malar J. 2021 Jul 6;20:305. doi: 10.1186/s12936-021-03837-5 (PMC8259116; doi:10.1186/s12936-021-03837-5)
Supplement: Supplementary file 1 — Additional file 1: The list of socioeconomic status (SES) variables. [file 12936_2021_3837_MOESM1_ESM.docx]

**The list of socioeconomic status (SES) variables**

| **No.** | **Variables** | **Type** | **Description** |
| --- | --- | --- | --- |
| 1. | Size of household | Continuous | Number of household members |
| 2. | Wall of house | Categorical | 1) Bamboo/ wood  2) Brick/concrete |
| 3. | Roof of house | Categorical | 1) Thatch roofs  2) Terracotta/concrete |
| 4. | Floor of house | Categorical | 1) Bamboo/ wood  2) Concrete |
| 5. | No vehicle in household | Categorical | 1) Yes  2) No |
| 6. | Bike in household | Categorical | 1) Yes  2) No |
| 7. | Motorcycle in household | Categorical | 1) Yes  2) No |
| 8. | Tractor in household | Categorical | 1) Yes  2) No |
| 9. | Car in household | Categorical | 1) Yes  2) No |
| 10. | No asset in household | Categorical | 1) Yes  2) No |
| 11. | Chicken in household | Categorical | 1) Yes  2) No |
| 12. | Pig or goat in household | Categorical | 1) Yes  2) No |
| 13. | Cow or horse in household | Categorical | 1) Yes  2) No |
| 14. | TV or refried in household | Categorical | 1) Yes  2) No |
| 15. | Elephant in household | Categorical | 1) Yes  2) No |
| 16. | Family income | Continuous | The total amount of money earned by every member of household |
| 17. | Number of room in household | Continuous | Number of rooms in household such as bedroom, living room, kitchen, bathroom, and a toilet |
| 18. | Education level of head of household | Categorical | 1) Illiterate  2) Literate |
| 19. | Ethnic of head of household | Categorical | 1) Thai  2) Karen |
| 20. | Occupation of head of household | Categorical | 1) House work  2) Other occupation such as agriculturalist, merchant, government official, and casual employed |
| 21. | Number of sleeping space in household | Continuous | Number of a separate space where any member (s) of the household regularly slept in one spot or in one bed net in household |
| 22. | Household member can speak Thai | Categorical | 1) Yes  2) No |

We used the variables in this table to construct a single socioeconomic score using Factor Analysis of Mixed Data (FAMD). FAMD was chosen rather than principal components analysis because we used a combination of continuous and categorical variables. The first dimension of the FAMD contributed to 19.7% of the overall variation and the second dimension contributed 9.8%. We ran models with both the first and second dimension included, but the model fit was best with only the first dimension (assessed through comparing the AIC and BIC values) and so we retained only the first dimension in the final model. The FAMD was done using R cran software version 4.0.2 and the FactoMineR package.
